# Two-wavelength infrared responsive hydrogel actuators containing rare-earth photothermal conversion particles

**DOI:** 10.1038/s41598-018-31932-2

**Published:** 2018-09-10

**Authors:** Satoshi Watanabe, Hiroshi Era, Masashi Kunitake

**Affiliations:** Faculty of Advanced Science and Technology, Kumamoto University, 2-39-1 Kurokami, Chuou-ku, Kumamoto City, Kumamoto, 860-8555 Japan

## Abstract

Two-wavelength infrared responsive soft actuators composed of rare-earth-oxide particles composited in a thermoresponsive hydrogel have been constructed. Because Nd_2_O_3_ and Yb_2_O_3_ particles possess independent narrow infrared adsorption at 808 and 980 nm, respectively, the vicinity of the particles in the gel can be individually heated by irradiation at each adsorption wavelength, inducing a local volume phase transition. The wavelength-selective volume phase transition can be controlled based on the combination of the particles incorporated in the gels and the wavelength of the irradiation laser at the optimized water temperature. Only the alternatively correct combinations successfully induced selective local clouding at the irradiation spots in the gel sheets. The original transparency of the gel was immediately recovered by turning off the light. Furthermore, rod-shaped block gels with Nd_2_O_3_ and Yb_2_O_3_ particles separately arranged on the left and right sides at the bottom of the rods were prepared to demonstrate wavelength-selective bending motion. The correct light combination caused reversible bending motion of only the side of the rod gel with the corresponding adsorbed particles.

## Introduction

Soft actuators have attracted much attention for application in artificial muscles and biomedical devices^1–3^. The various driving principles of actuators include an electric response using an electrostatic force^4–8^ and the electrolyte movement^9–11^, an air pressure response using expansion of silicone elastomers^12–14^, a temperature response using a volume phase transition of a hydrogel or thermal expansion of an elastomer owing to ambient temperature^15–17^, a material response using hydrogel shrinkage owing to the ionic strength^18^ and solvent solubility^19^, an autonomous oscillation response using the Belousov–Zhabotinsky reaction^20^, and a photoresponse using the free volume change of the actuator induced by light absorption^19–33^.

For photoresponsive actuators, photoisomerization^21–23^, photoradiation forces^24^, and photothermal conversion^25–35^ have been used for remote control of the actuators by light irradiation. Based on photoisomerization by ultraviolet (UV) and visible light, the free volume change owing to isomerization of the photosensitive units, such as azobenzene and diarylethene, in the polymeric matrix directly induces a macroscopic shape change of the material.

Recently, near-infrared-light-controlled actuators have attracted much attention owing to their potential biological and medical applications, because near-infrared light transmits through living tissue^36^. A near-infrared-light-induced volume phase transition of thermosensitive poly(*N*-isopropylacrylamide) (PNIPAAm) hydrogels without a photosensitizer has been achieved by the direct heating of water^37^ and light radiation forces^24^. For a more efficient infrared-light-induced volume phase transition, PNIPAAm hydrogels incorporating photosensitizers (photothermal conversion materials) have been developed. Core/shell nanoparticles (Au^38^ and Au_2_S^39^ with a silica core) have been applied as photothermal conversion materials because of their high absorption cross-sections in the near-infrared region. Drug delivery systems^40^ and thermal therapy^41^ for treatment of cancer cells using hydrogels with core/shell nanoparticles have been proposed. Ahir and Terentjev^25^ and Mori and co-workers^26^ proposed near-infrared response actuators based on photothermal conversion materials (carbon nanotubes and gold nanorods) in silicone elastomers and PNIPAAm hydrogels, respectively. Various photothermal response actuators have been developed using a combination of carbon materials and thermosensitive polymers^27–35^. Local shape changes at desired points of the actuators have also been achieved by selectively focused light irradiation^33^.

In this study, we investigated two-wavelength infrared responsive soft actuators composed of thermoresponsive PNIPAAm hydrogels containing rare-earth-oxide (REO) particles as a photothermal conversion unit. A wavelength-selective volume phase transition can be achieved by the combination of the narrow band infrared adsorption of Nd_2_O_3_ and Yb_2_O_3_ particles at 808 and 980 nm, and the irradiation laser wavelength.

## Results and Discussion

Nd_2_O_3_ and Yb_2_O_3_ particles with diameters of 100 nm were used as wavelength-selective photothermal conversion REO materials at 808 (^4^F_9/2_–^2^H_9/2_ and ^4^F_9/2_–^4^F_5/2_ transitions of Nd^3+^) and 980 nm (^2^F_7/2_–^2^F_5/2_ transition of Yb^3+^), respectively^42,43^. Rare-earth nanomaterials, including REOs, are frequently used as fluorescence, phosphorescence, and upconversion luminescence emission materials, and the emission efficiency is then crucial for the required performance^44–46^. Conversely, for photothermal conversion applications, the excited energy should be released by thermal radiation, but not by light emission. Pure REO particles with a relatively small particle size possess almost no fluorescence.

Plate- and rod-shaped PNIPAAm gels containing REO particles were fabricated by photoradical polymerization in a glass plate gap and a capillary, respectively^16^. The lower critical solution temperature (LCST) of the gels used in the experiments was about 36.5 °C, which is higher than that of the homopolymers (Figure S1). Although the LCST changed with the type of cross-linker and composition ratio, the polymer gels with the same composition ratio had approximately the same LCST. In addition, introduction of REO particles into the gels had almost no effect on the LCST. To confirm the stable performance of the thermal actuators, the REO-particle-incorporated gels were shrunk and swelled by heating over and cooling under the LCST, respectively.

To induce the volume phase transition based on photothermal conversion, many factors in addition to the irradiation wavelength should be considered, such as the light irradiation intensity, rare-earth particle concentration, and water temperature of the gel. Among these factors, the gel temperature (the temperature of the water immersing gel) is an important parameter and needs to be delicately controlled. When the gel temperature is too low, very high laser intensity is required to overcome heat dissipation for local heating above the LCST. Conversely, when the gel temperature is too close to the LCST, an undesirable volume phase transition might occur and wavelength selectivity cannot be achieved.

**Figure 1:**
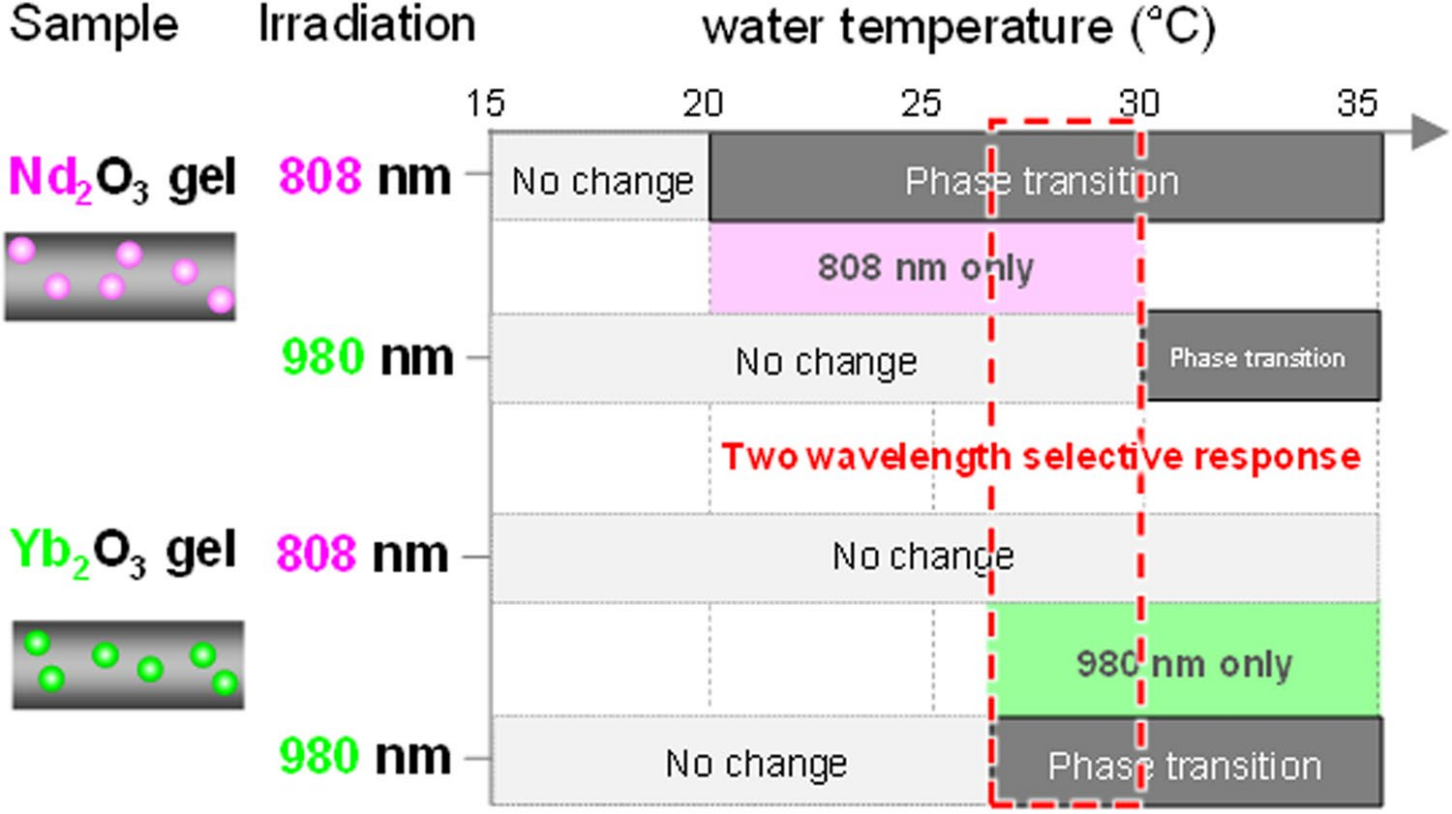
Near-infrared-light-induced volume phase transition–temperature diagrams for 1 mm-thick Nd_2_O_3_ and Yb_2_O_3_ plate gels irradiated with 808 and 980 nm light at 3 W cm^−2^ for 60 s, respectively. The phase transition temperatures were visually determined by the cloudiness of the irradiated areas.

The volume phase transition induced by near-infrared irradiation was investigated as the function of the gel (water) temperature using the REO-particle-dispersed plate gels (ca. 1 mm thickness) placed in Petri dishes containing water. The volume phase transition induced by light was judged by the visual turbidity change of the irradiation area of the sample. Figure 1 shows the temperature diagrams of the clouding responses of the gels induced by 808 or 980 nm laser irradiation at 3 W cm^−2^ for 60 s.

The Yb_2_O_3_ gel showed a volume phase transition induced by 980 nm light irradiation in the temperature range 27–35 °C. The light intensity at 980 nm was not sufficient to induce a volume phase transition in the Yb_2_O_3_ gel below 27 °C. Above 35 °C, spontaneous clouding owing to the start of the volume phase transition occurred without light irradiation. Because of the absence of an absorption band at 808 nm, 808 nm light irradiation did not cause a volume phase transition in the Yb_2_O_3_ gel.

For the Nd_2_O_3_ gel, 980 nm light irradiation also resulted in a volume phase transition above 30 °C despite Nd_2_O_3_ not having an absorption band at 980 nm. The volume phase transition under 980 nm light irradiation is because of the slight overlap of water adsorption at 980 nm. The influence of direct heating of water by 980 nm light irradiation was ignored below 30 °C. Because 808 nm light irradiation induced a transition in the Nd_2_O_3_ gel above 20 °C, the 808 nm light-induced phase transition could be achieved in the temperature range 20–30 °C. Therefore, the overlapping water temperature region from 27 to 30 °C is a suitable temperature range for the wavelength-selective volume phase transition in the Nd_2_O_3_ and Yb_2_O_3_ gels.

**Figure 2:**
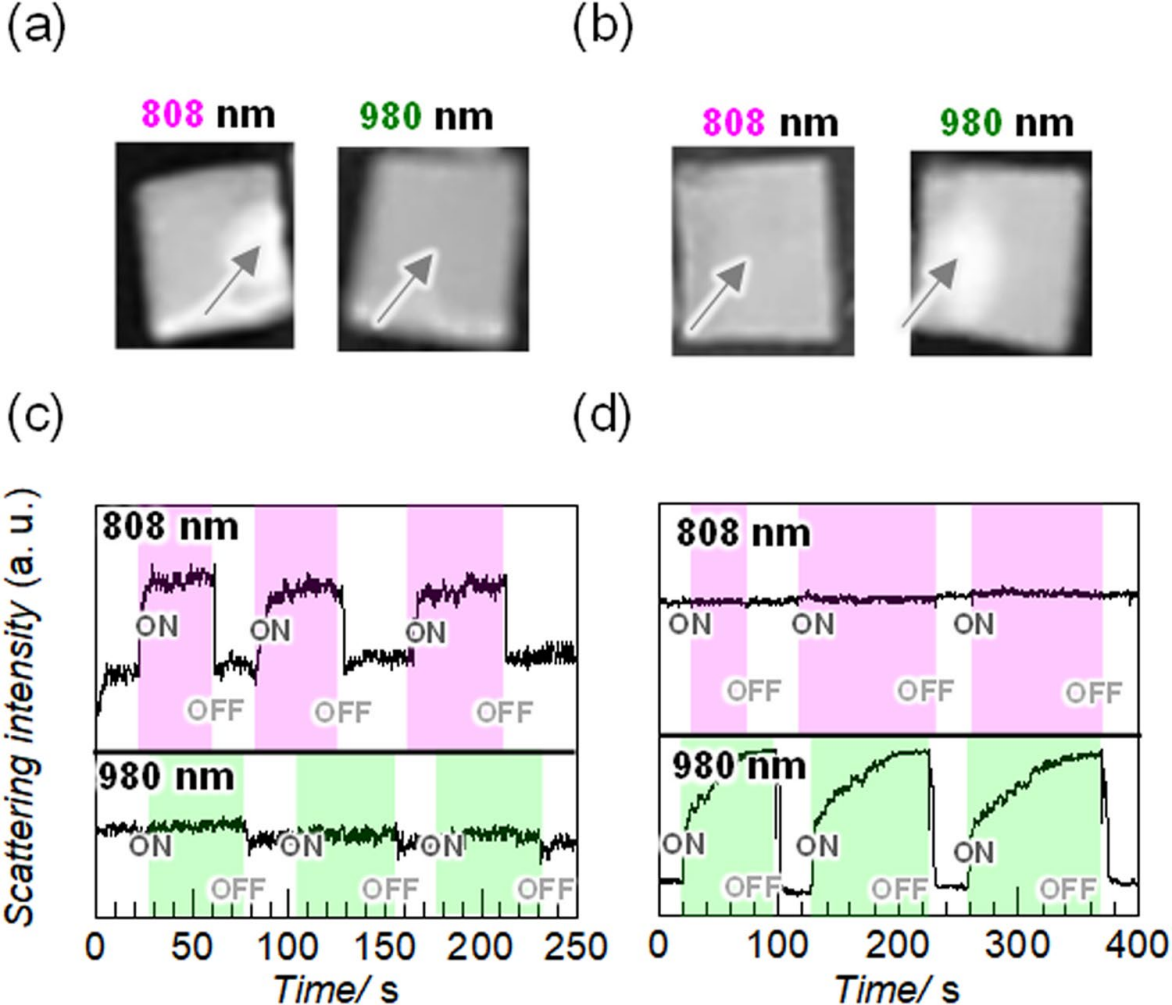
(**a**,**b**) Photographs and (**c**,**d**) relative turbidity changes of the Nd_2_O_3_ plate gels (**a** and **c**) and Yb_2_O_3_ plate gels (**b** and **d**) in water at 28 °C under intermittent irradiation with 808 and 980 nm light at 3 W cm^−2^. The dimensions of the plate gels are 5 mm × 5 mm with a thickness of about 1 mm. The turbidity changes were measured with 532 nm laser light at 1 mW. The arrows indicate the centres of the irradiation spots.

Figure 2 shows the turbidity changes at the irradiation points on the gels by “ON/OFF” laser irradiation at a gel temperature of 28 °C. The volume phase transition was observed as the change of the scattering intensities of the samples using a 532 nm probe laser. As expected, 980 nm light irradiation on the Nd_2_O_3_ gel caused no response, but 808 nm light irradiation produced a white turbid spot, as shown in Fig. 2a. With 808 nm light irradiation, the scattering intensity at the irradiated point instantly increased and reached a certain saturation level within several seconds, as shown in Fig. 2c. When the light was turned off, the scattering intensity rapidly decreased and returned to the original value. The thermal energy at the irradiation spots promptly dissipated after the laser was turned off. This indicates that the constant turbidity under appropriate laser irradiation is regulated by the thermal equilibrium between heating by irradiation and thermal scattering.

As shown in Fig. 2b and d, the Yb_2_O_3_ gel showed the opposite responses: no response with 808 nm light irradiation and a turbid spot with 980 nm light irradiation. In addition, the scattering intensity of the Yb_2_O_3_ gel increased with 980 nm light irradiation but not with 808 nm light irradiation. About 100 s irradiation was required for saturation of the scattering intensity, which is longer than that required for the Nd_2_O_3_ gel. This might be because of loss of the 980 nm laser power by absorption of water. When the light was turned off, the scattering intensity rapidly decreased and returned to the original value, similar to the case of the Nd_2_O_3_ gel. The Nd_2_O_3_ and Yb_2_O_3_ gels showed good reversibility and reproducibility with repeated light irradiation. No damage of the gels was observed by laser light irradiation for less than 20 min and the gels were almost perfectly recovered in terms of scale and turbidity after turning off the light irradiation.

**Figure 3:**
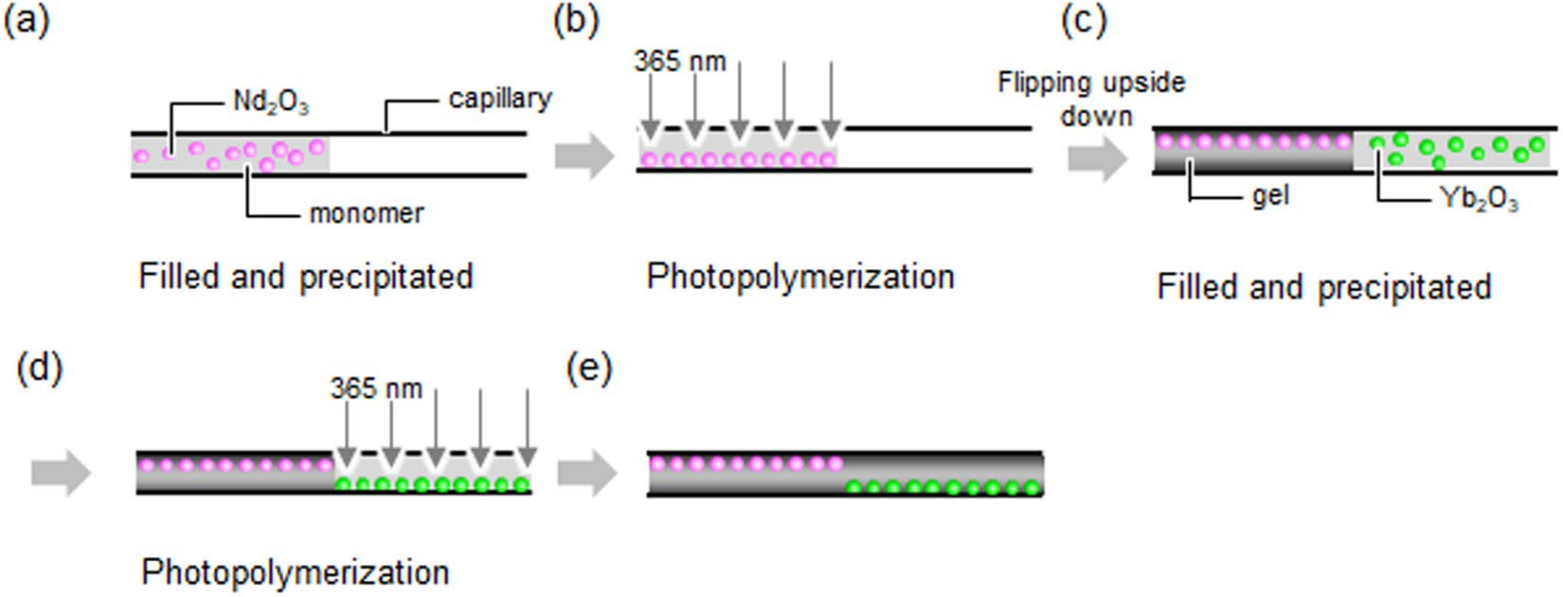
Schematic of fabrication of REO-particle-precipitated rod gels for bending motion. Fabrication of the (**a**,**b**) Nd_2_O_3_-particle–gel moiety, (**c**,**d**) Yb_2_O_3_-particle–gel moiety, and (**e**) final rod gels with Nd_2_O_3_- and Yb_2_O_3_-particle-precipitated moieties.

To convert the volume phase transition into bending motion, PNIPAAm block-rod gels with diameters of 0.1, 0.6, and 1.0 mm consisting of a Nd_2_O_3_ gel moiety and a Yb_2_O_3_ gel moiety were prepared, as shown in Fig. 3. In each REO block-rod gel moiety, the REO particles heterogeneously distributed on the bottom of the rod by sedimentation. Before photopolymerization, the Nd_2_O_3_ particle dispersion containing the monomers was injected into glass capillaries until they were half full, and the capillaries were then allowed to stand until the Nd_2_O_3_ particles settled to the bottom. After photopolymerization of the Nd_2_O_3_ gel, the Yb_2_O_3_-particle-precipitated rod gel was fabricated on the opposite side of the capillary in a similar manner. The REO particles precipitated on the bottom of the rods induced light heating on one side of the rods, resulting in directional bending of the rods. The Nd_2_O_3_ and Yb_2_O_3_ rod gels connected to form one rod gel were visually recognizable. From hand pulling of the rods, the connected part of the two rod gels had approximately the strength as the bulk parts. The rod gels were set in Petri dishes with water at 28 °C, and 808 and 980 nm laser light was then irradiated at each of the Nd_2_O_3_ and Yb_2_O_3_ sides of the rod gel for visual observation of bending.

**Figure 4:**
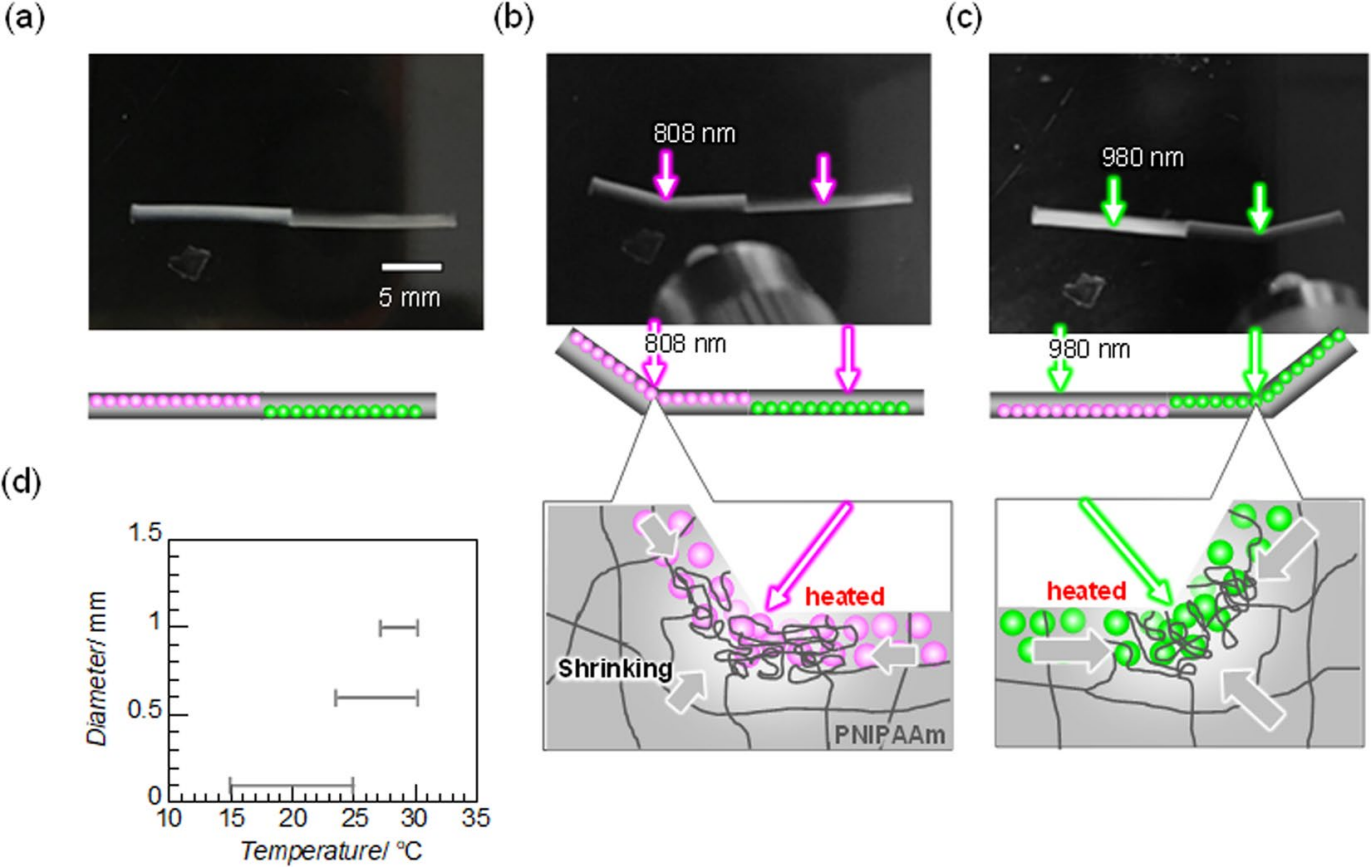
Photographs of a REO-particle-precipitated rod gel with (**a**) no irradiation, and (**b**) 808 and (**c**) 980 nm near-infrared light irradiation at 3 W cm^−2^ for 60 s at 28 °C. The left and right sides of the 1-mm-diameter rod gel are the Nd_2_O_3_- and Yb_2_O_3_-particle-precipitated gel moieties, respectively. For ease of photographing, the position of the settled REO particle part in the rod gel in (**c**) was inverted with respect to that in (**a**) and (**b**). (**d**) Temperature range for the selective-bending response plotted against the rod diameter.

Figure 4 shows a typical demonstration of wavelength-selective bending of a rod gel (1.0 mm diameter). As shown in Fig. 4b and c, bending of the rod gel by about 30° occurred at the irradiation points for both sides as a photosteady state when the Nd_2_O_3_ and Yb_2_O_3_ moieties were irradiated at 808 and 980 nm, respectively. Irradiation with the correct wavelength light led to bending of any part of the gels. The irradiation point immediately started to turn turbid white. The rod gel then began to bend and reached a steady bending state in less than 10 s, which is longer than the clouding response time of the REO-particle-dispersed plate gels. When the irradiation was turned off, the white turbidity promptly disappeared and the gel rod gradually returned to its original shape within several minutes. The opposite combination of absorption band mismatch showed no change, even for irradiation for several minutes. Niidome *et al*.^26^ reported the shrinking processes of PNIPAAm gels with gold nanorods based on photothermal conversion.

The 0.1- and 0.6-mm-diameter rod gels showed essentially the same wavelength-selective bending responses with light irradiation as the 1-mm-diameter rod gel. The real bending motion of the 0.6-mm-diameter rod gel is shown in Figure S2 and Movie S1. For the 0.6- and 0.1-mm-diameter rod gels, the bending angles were approximately 45° and 60°–180°, respectively. The bending angle in each photosteady state increased with decreasing diameter of the rod gel. The narrow rod (0.1 mm diameter) showed low reproducibility in terms of the bending performance owing to the difficulty in controlling the concentration and distribution of REO particles. The bending and returning speeds also increased with decreasing diameter, as with ordinary gels^46,47^. The returning speed of the narrow rod was exceptionally slow, probably because of the uncontrolled gel structure.

For all of the rod gels, both sides of the gel rods underwent irradiation-induced bending towards the side with the particles that undergo a volume phase transition at the irradiated light wavelength, indicating that shrinkage on the side containing those particles was larger than that on the opposite side (Fig. 4b and c). This indicates that the effective heat to induce shrinkage was not transferred to the entire cross-section of the rod. When the whole rod was simply heated above the LCST, the rod shrank towards the side without the particles. Akashi *et al*.^16^ prepared NIPAM sheets with silica nanoparticles attracted close to one side by electrophoresis. The nanoparticle-gradient sheets bent towards the side of the sheets with lower nanoparticle concentration, and the porous-gradient sheets prepared by removing the nanoparticles bent towards the porous side.

Figure 4d shows the suitable temperature ranges for the two-wavelength selective responses of rods with different diameters. With decreasing rod diameter, the lower temperature limit of the selective response decreases and the temperature range of the selective response increases. Local heating by laser irradiation is easier for the selective response of rods with a smaller diameter, although the composition ratio of the REO particles and NIPAAm monomers is constant and thermal diffusion of the small rod is expected to be faster. In addition, the upper bound of the temperature range for the rod with the smallest diameter of 0.1 mm is the lowest. This might be because of the ease of heating water by 980 nm irradiation.

We also prepared 0.1-mm-thick sheet-shaped gels. Nd_2_O_3_ and Yb_2_O_3_ particles were separately precipitated on the right and left sides of the sheets by stepwise sedimentation, similar to the REO rods. Wavelength-selective shrinking and bending of the films were observed at the irradiation spots, as shown in Figure S3.

## Conclusions

The two-wavelength response gel system containing narrow absorption band photothermal conversion materials reported here introduces a new concept for soft actuators to achieve multiple local actions towards construction of micro- or nanoscale robots and devices. This concept can be generalized to design and construct various multifunction remote control soft actuators through the use of a wide range of rare-earth materials. Because rare-earth infrared adsorption materials are not limited to the REO particles investigated here, this method can be readily applied to arbitrary elements, complex molecules, and nanoparticles.

In contrast to popular photothermal conversion materials with wide adsorption bands, the wavelength selectivity of rare-earth materials is excellent because of narrow absorption and the small influence of the chemical environment on the absorption position. It should be mentioned that there is a trade-off between the wavelength selectivity and the photothermal conversion efficiency because narrow absorption bands are disadvantageous in terms of effective photothermal conversion compared with broad absorption band materials. In addition, because the electrons in the highest occupied molecular orbital level are present in the inner shell orbit, the molar extinction coefficient is lower than that of typical photothermal conversion materials.

Microactuators with multiple actions from sub-micrometres to several tens of micrometres can be fabricated by conventional photolithography, soft lithography, inkjet/three-dimensional printing, and so forth. The results reported here pave the way for “top-down” design and construction of a vast range of noncontact remote micromachines incorporating a variety of actuator elements that are responsive to different wavelengths. The potential applications of the systems are wide, including biological and medical applications^48^ using “near-infrared windows”, remote control of the valves of microflow systems, sub-micrometre-scale mass transportation systems, and self-propelling micromachines.

## Methods

### Preparation of the homogeneously dispersed REO particle plate gels

The glass plates were ultrasonicated in acetone and chloroform for 20 min each and exposed to an UV-ozone atmosphere generated by an UV/ozone cleaner (PL-110, Sen Light Corp., Osaka, Japan) for 10 min. For the monomer solution, a 0.7 M NIPAAm aqueous solution (typically 3–4 mL) containing 0.035 M *N*′-methylene bisacrylamide (5 mol% with respect to NIPAAm) and 0.007 M 2,2-diethoxy acetophenone (1 mol% with respect to NIPAAm) was used. Nd_2_O_3_ (*d* = 7.24 g cm^−3^) or Yb_2_O_3_ (*d* = 9.17 g cm^−3^) particles were dispersed in the solution at 8 mg mL^−1^ (0.110 vol%, and 0.0872 vol%, respectively) by ultrasonication. These REO-particle dispersions were filled between two glass plates with 1 mm spacers and the samples were immediately exposed to UV light for 60 min at 4 °C to form the gels before particle precipitation. The 360 nm UV light at 80 mW cm^−2^ was generated by a light source (BOX S3000, Sunhayato Corp., Japan). After polymerization, the plate gels were removed from the gaps between the glass plates, and the REO gels were stored in water prior to use. The swelling degrees of the gels were about 5 wet-g/dry-g.

### Turbidity measurements of the homogeneously dispersed REO particle plate gels

The scattering intensities of the gels were measured with respect to the irradiation time of 808 and 980 nm near-infrared light at 3 W cm^−2^ generated by LSR808NL-FC-3W and LSR980NL-FC-3W laser light sources (NaKu Technology Co., Ltd., China) in water at 28 °C and probed by 532 nm laser light with power of 1 mW generated by a PR10-GC CP laser light source (Cannon, Japan), as shown in Figure S4. The 532 nm probe light was detected through a 550 ± 40 nm band-pass filter by an OP-2 photocurrent-type semiconductor sensor connected to a FieldMaxII-TO controller (Coherent Japan Corp., Japan). The distance between the sample and the laser was about 1 cm and the light spot area on the sample was 1 cm^−2^.

### Preparation of the heterogeneously precipitated REO particle block-rod gels

The block-rod gels containing locally arranged REO particles were prepared by step-wise polymerization in glass capillaries (Fig. 3). Glass capillaries with diameters of 0.2, 0.6, and 1 mm were half filled with a Nd_2_O_3_-particle aqueous dispersion (8 mg mL^−1^) containing 0.7 M NIPAAm, 0.035 M (5 mol%) *N*,*N*′-methylenebisacrylamide, and 0.007 M 2,2-diethoxyacetophenone (1 mol%). The samples were photopolymerized by exposure to UV light at the same conditions as above. Prior to polymerization, the capillaries were allowed to stand for 24 h to precipitate Nd_2_O_3_ particles on the bottom of the capillaries by gravitational force. The empty space next to the Nd_2_O_3_-rod gels in the capillaries was then filled with a Yb_2_O_3_-particle dispersion (8 mg mL^−1^) containing the same monomer precursors. The capillaries were rotated for the Yb_2_O_3_ particles to precipitate on the same or opposite side to the Nd_2_O_3_ particles. After precipitation of the Yb_2_O_3_ particles for 24 h, photopolymerization of the samples was performed. The block-rod gels were shrunk by heating at 50 °C and then taken out of the capillaries.

### Actuator operation of two-wavelength-responsive rod hydrogels

The block-rod gels were placed in glass dishes containing 1 cm of water at the optimal temperatures. They were then exposed to 808 nm or 980 nm light at 3 W cm^−2^ to induce bending actuation. The distance between the sample in the water and the 808 or 980 nm laser was about 1 cm and the light spot area on the sample was 1 cm^−2^.

## Electronic supplementary material

Movie of actuator

Supplementary information
