# Supplementary material for: Two-wavelength infrared responsive hydrogel actuators containing rare-earth photothermal conversion particles
Source: Sci Rep. 2018 Sep 10;8:13528. doi: 10.1038/s41598-018-31932-2 (PMC6131526; doi:10.1038/s41598-018-31932-2)
Supplement: Supplementary file 2 — Supplementary information [file 41598_2018_31932_MOESM2_ESM.docx]

Supporting Information

Two-wavelength infrared responsive hydrogel actuators containing rare-earth photothermal conversion particles

Satoshi Watanabe*, Hiroshi Era, and Masashi Kunitake*

Faculty of Advanced Science and Technology, Kumamoto University, 2-39-1 Kurokami, Chuou-ku, Kumamoto City, Kumamoto 860-8555, Japan.

*watasato@kumamoto-u.ac.jp (S.W.) and kunitake@kumamoto-u.ac.jp (M.K.)

**Figure S1.** Transmittance and relative volume change of the REO-particle-dispersed PNIPAAm hydrogels plotted against the water temperature. The water temperature change was 1 °C min^−1^. The transmittance was measured with 532 nm probe light by a UV–visible spectrometer.

**Figure S2.** Photographs of **a** REO-containing rod gels with a rod diameter of 0.6 mm in water at 28 °C. Both of Nd_2_O_3_ and Yb_2_O_3_ particles were precipitated on the left and right sides of the same bottom of the rods. Near-infrared light at **b** 808 nm and **c** 980 nm was irradiated at 3 W cm^−2^ for 60 s. Please also see **Movie S1**.

**Figure S3.** Photographs of **a** REO-containing sheet gels with a thickness of 0.1 mm in water at 24 °C. Both of Nd_2_O_3_ and Yb_2_O_3_ particles were precipitated on the left and right sides of the same bottom of the rods. Near-infrared light at **b** 808 nm and **c** 980 nm was irradiated at 3 W cm^−2^ for 60 s.

**Figure S4.** Illustration of turbidity measurements of REO-particle homogeneously dispersed plate gels in water with a 532 nm probe at 1 mW detected by a photocurrent sensor through a band path filter of 550 nm ± 40 nm. Near infrared light at 808 nm and 980 nm was irradiated at 3 W cm^-2^ and at an irradiated spot size of 1 cm^2^.
